# Stand-Alone Posterior Expandable Cage Technique for Adjacent Segment Degeneration with Lumbar Spinal Canal Stenosis: A Retrospective Case Series

**DOI:** 10.3390/medicina57030237

**Published:** 2021-03-04

**Authors:** Woo-Jin Choi, Seung-Kook Kim, Manhal Alaraj, Hyeun-Sung Kim, Su-Chan Lee

**Affiliations:** 1 Department of Neurosurgery, Spine Center, Hurisarang Hospital, 618 Gyeryong-ro, Seo-gu, Daejeon 35299, Korea; neurocwj@hanmail.net; 2 Himchan and UHS Spine and Joint Centre, Neurosurgery, University Hospital Sharjah, University Street 1, Sharjah 72772, United Arab Emirates; manhal.Alaraj@uhs.ae; 3 Department of Pharmaceutical Medicine and Regulatory Sciences, College of Medicine and Pharmacy, Yonsei University, 85, Songdokwahak-ro, Yeonsu-gu, Incheon 21983, Korea; 4 Joint and Arthritis Research, Orthopaedic Surgery, Himchan Hospital, 20, Sinmok-ro, Yangcheon-gu, Seoul 07999, Korea; scleeos@empas.com; 5 Department of Orthopedic Surgery, University Hospital Sharjah, University Street 1, Sharjah 72772, United Arab Emirates; 6 Department of Spine Center, Gangnam Nanoori Hospital, Gangnam, Seoul 06048, Korea; neurospinekim@gmail.com

**Keywords:** adjacent segment degeneration, proximal junctional kyphosis, expandable interbody cage, minimally invasive spine surgery, subsidence

## Abstract

*Background and Objectives*: Symptomatic adjacent segment degeneration (ASD) with lumbar spinal canal stenosis (LSCS) is a common complication after spinal intervention, particularly interbody fusion. Stand-alone posterior expandable cages enable interbody fusion with preservation of the previous operation site, and screw-related complications are avoided. Thus, the aim of this study was to investigate the clinicoradiologic outcomes of stand-alone posterior expandable cages for ASD with LSCS. *Materials and Methods*: Patients with persistent neurologic symptoms and radiologically confirmed ASD with LSCS were evaluated between January 2011 and December 2016. The five-year follow-up data were used to evaluate the long-term outcomes. The radiologic parameters for sagittal balance, pain control (visual analogue scale), disability (Oswestry Disability Index), and early (peri-operative) and late (implant) complications were evaluated. *Results*: The data of 19 patients with stand-alone posterior expandable cages were evaluated. Local factors, such as intervertebral and foraminal heights, were significantly corrected (*p* < 0.01 and *p* < 0.01, respectively), and revision was not reported. The pain level (*p* < 0.01) and disability rate (*p* < 0.01) significantly improved, and the early complication rate was low (*n* = 2, 10.52%). However, lumbar lordosis (*p* = 0.62) and sagittal balance (*p* = 0.80) did not significantly improve. Furthermore, the rates of subsidence (*n* = 4, 21.05%) and retropulsion (*n* = 3, 15.79%) were high. *Conclusions*: A stand-alone expandable cage technique should only be considered for older adults and patients with previous extensive fusion. Although this technique is less invasive, improves the local radiologic factors, and yields favorable clinical outcomes with low revision rates, it does not improve the sagittal balance. For more widespread application, the strength of the cage material and high subsidence rates should be improved.

## 1. Introduction

Adjacent segment degeneration (ASD) with lumbar spinal canal stenosis (LSCS) is one of the main complications after spinal instrumentation, occurring in 5.2–18.2% of patients [1,2]. ASD with LSCS can markedly impair a patient’s quality of life, with the narrowing of the upper or lower spinal canal and the malalignment of sagittal balance causing back- and leg-related symptoms [3]. Accordingly, several surgical treatments for this condition have been attempted, including (1) additional decompression [4]; (2) decompression and posterolateral fusion (PLF) [4]; (3) direct spinal decompression, including posterior lumbar interbody fusion (PLIF) or transforaminal lumbar interbody fusion (TLIF) with pedicle screw fixation (PSF) [5]; (4) indirect decompression, including anterior lumbar interbody fusion (ALIF) or lateral lumbar interbody fusion (LLIF) with PSF [4]; and (5) extensive correction of sagittal balance and various types of fusion [6]. Although decompression is minimally invasive, it only provides temporary symptom relief, and the long-term follow-up outcomes are not favorable [4].

PLIF, TLIF, ALIF, and LLIF are considered the mainstays of treatment for ASD. However, they require destruction of the musculature and replacement of the previously implanted screw. Furthermore, they are associated with a higher risk of complications than the initial fusion surgery [4,5]. Extensive correction of sagittal balance shows long-term effectiveness, but its application is limited by the aggressive nature of the procedure and failure of instrumentation [6]. Meanwhile, stand-alone posterior expandable cages are less invasive and can simultaneously decompress the spinal canal and restore lumbar lordosis (LL) [7,8]. However, to the best of our knowledge, the stand-alone posterior expandable cage technique has only been evaluated as an initial procedure and not as a treatment for ASD with LSCS. Additionally, a high rate of subsidence due to a lack of posterior fixation has been highlighted as a disadvantage [9]. Thus, the aim of this study was to evaluate the clinicoradiologic outcomes of stand-alone posterior expandable cages for ASD with LSCS. It was found that posterior standing cages were less invasive, improved local radiologic factors, and yielded favorable clinical outcomes. However, this technique did not improve spinopelvic parameters.

## 2. Materials and Methods

### 2.1. Study Design and Patients

This single-center retrospective case series was approved by the ethics committee of Hurisarang Hospital, Daejeon, Korea (Himchan IRB 2019-06, 21 December 2019 approved) and was conducted in accordance with the ethical standards in the 1964 Declaration of Helsinki and its later amendments. Informed consent was obtained from all patients.

This study was performed between January 2011 and December 2016 at Hurisarang Hospital, Daejeon, Korea. The subjects were patients with persistent neurologic symptoms and radiologically confirmed ASD with LSCS. The inclusion criteria were as follows: (1) history of previous surgical intervention (PLF, lumbar interbody fusion, or polymethylmethacrylate-based acrylic bone cement augmentation); (2) radiologically confirmed ASD with LSCS (cross-sectional area of the dural sac < 100 mm [2], bilateral exiting or traversing nerve root compression, or the presence of redundant nerve in the spinal canal); (3) relapse of back and leg pain after at least 2 months of improvement after primary surgery (neurogenic intermittent claudication < 30 min or a distance of 200 m, unresponsive to medical treatments); and (4) available follow-up data of at least 5 years. The exclusion criteria for assisted screw fixation were spondylolisthesis (higher than grade II) and severe osteoporosis (bone density ≥ 1.8 standard deviations below the mean for young adults).

### 2.2. Surgical Protocol

**Figure 1:**
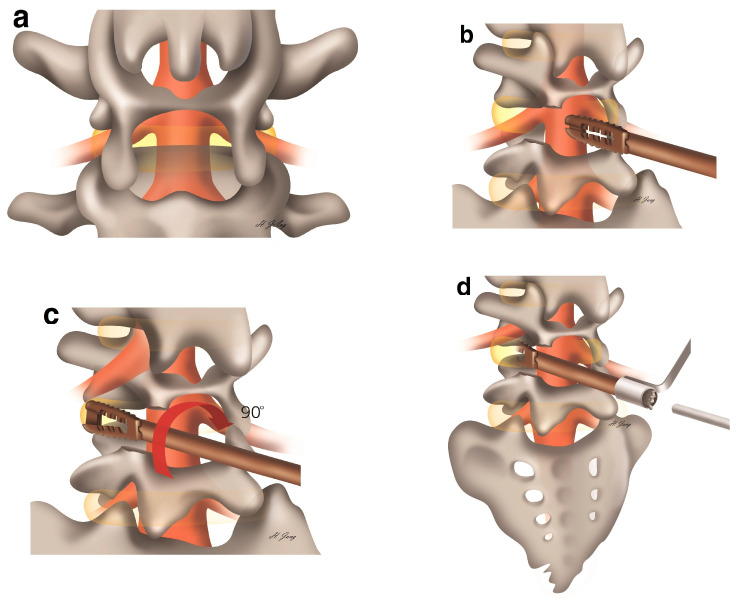
Surgical procedure for stand-alone expandable cages. After discectomy and endplate preparation (**a**). The cage is inserted with the patient in a supine position (**b**). The cage is rotated 90° to restore lumbar lordosis (**c**). Lastly, bone chips are inserted using a specially designed injector (**d**).

The surgical intervention was performed under epidural or spinal anesthesia. After prone positioning on a radiolucent table, portable radiography was used to confirm the operative level. Alcohol and betadine dressing were applied aseptically. One level of ASD needed a 5 cm incision. First, bilateral multifidus muscle dissection with monopolar coagulator (Figure 1a) and facet preserving laminectomy with an automated drill and Kerrison punches were performed (Figure 1b). At least half of the facet was preserved for interbody fusion. Second, total discectomy and endplate preparation were performed with a shaver and pituitary forceps. Third, the reclined stand cages were inserted and rotated 90° until LL was achieved (Figure 1c). Fourth, autologous and allograft bone chips were inserted using a bone pusher (Figure 1d). This procedure was repeated bilaterally. Finally, the muscle was sutured with an absorbable suture and the skin was stapled. The patient was placed on bed rest until postoperative day (POD) 3. Laboratory tests were conducted on POD 3 and 7, and magnetic resonance imaging was performed on POD 7. Thoracolumbar sacral orthosis was applied 3 months after the procedure. Plain radiography was performed on POD 3; after 2, 6, and 12 months; and after 60 months at the last follow-up.

### 2.3. Outcome Measures

**Figure 2:**
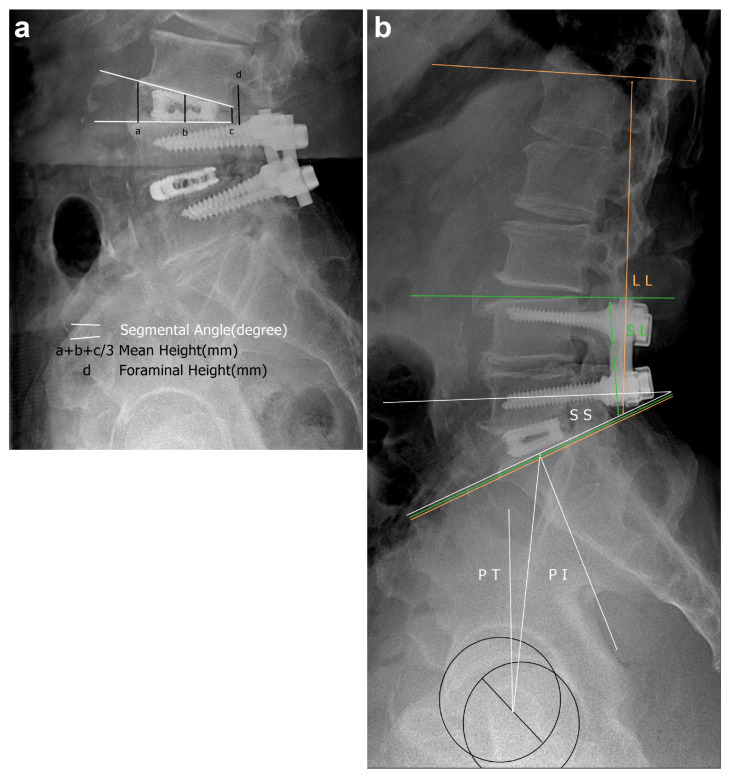
Radiologic evaluation of stand-alone cages. Anterior, middle, and posterior disc height, foraminal disc height, and segmental angle measurements (**a**). Sacropelvic profile and lumbar lordosis. Abbreviations: PT, pelvic tilt; PI, pelvic incidence; SS, sacral slope; SL, short lordosis; LL, lumbar lordosis (**b**)**.**

Using lateral plain radiography, the average lengths of the anterior, middle, and posterior disc spaces and foraminal height were evaluated as local factors. The segmental angle was checked using Cobb’s method (Figure 2a). Regarding lumbar factors, the Cobb angles of the upper endplate of the upper vertebral body and the lower endplate of the lower vertebral body (short lumbar lordosis (SL)) and the Cobb angles of the upper endplate of the first lumbar vertebra and the upper endplate of the sacrum (LL) were checked. With respect to the sacropelvic profile, pelvic incidence, pelvic tilt, and sacral slope (Figure 2b) were evaluated. Preoperative and final follow-up lateral radiography findings were also compared. As for clinical factors, leg radiating pain was assessed using the visual analogue scale (VAS), while quality of life modification was evaluated using the Oswestry Disability Index. Safety was evaluated according to perioperative complications, revision, and late complications (retropulsion, subsidence, pseudoarthrosis, cage breakage, and additional ASD) at the final follow-up. Clinical data were recorded via a questionnaire during hospitalization, and radiologic evaluation was performed by spine surgery specialists with more than 10 years of experience (SK and WC).

### 2.4. Statistical Analysis

Continuous variables were compared using the univariate t-test and Mann–Whitney test, whereas categorical variables were examined using the chi-square test. All statistical analyses were performed using R software for Windows version 3.6.1 (R Foundation for Statistical Computing, Vienna, Austria). A *p*-value < 0.05 was considered statistically significant.

## 3. Results

### 3.1. Patient Characteristics

**Figure 3:**
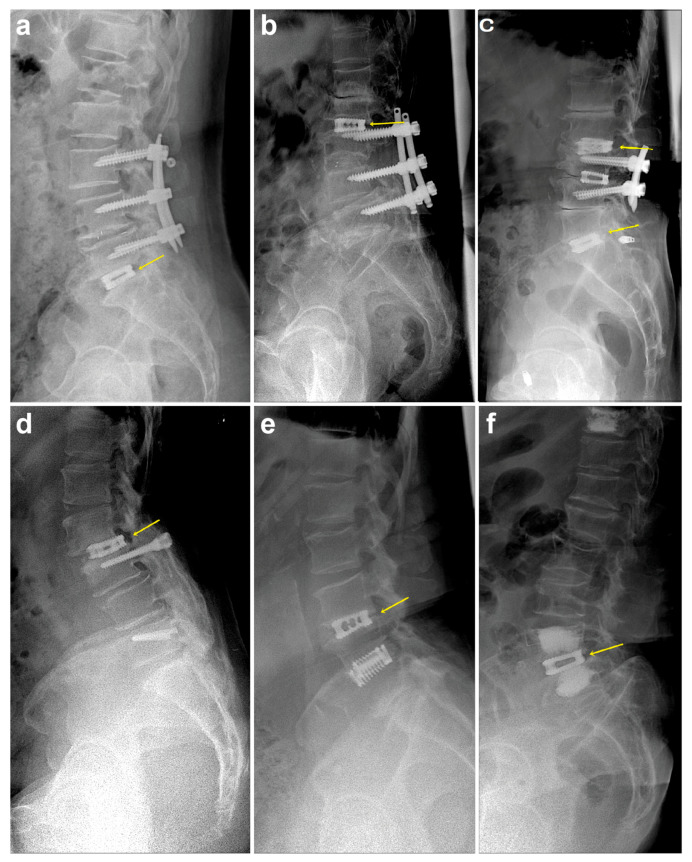
Application of stand-alone cages for various levels of adjacent segment degeneration (ASD). Lower-level ASD (**a**), upper-level ASD (**b**), upper- and lower-level ASD (**c**), after posterolateral fusion (**d**), after previous stand-alone cage surgery (**e**), and after percutaneous vertebroplasty (**f**).

Of the 33 patients with operative ASD with LSCS (Table 1), 7 were treated with additional fusion and screw extension, 4 did not respond to the follow-up phone call, and 3 did not respond to the Oswestry Disability Index questionnaire (response rate, 82.60%). Thus, 19 patients (63.16% females) with a mean age of 67.13 ± 9.31 years were included in the analysis. The main ASD indication was sensorimotor symptoms (*n* = 11, 57.89%). Furthermore, four patients (21.05%) showed bladder/bowel symptoms. The predisposing factors for previous surgery were PLF (21.05%), PLIF (47.37%), stand-alone cages (26.32%), and percutaneous vertebroplasty (PVP) (5.26%) for lower-level ASD (Figure 3a), upper-level ASD (Figure 3b), and both upper- and lower-level ASD (Figure 3c) after PLIF, after PLF (Figure 3d), after previous single cage (Figure 3e), and after PVP (Figure 3f), respectively. The mean duration of ASD and follow-up was 8.26 years and 7.61 years, respectively. Radiologically severe degeneration (Pfirrmann disc degeneration grade 5) was identified in 68.42% (*n* = 13) of the patients.

### 3.2. Outcomes

The radiologic outcomes are summarized in Table 2. With respect to the local factors, the anterior, middle, and posterior disc and foraminal heights were significantly restored after 1 year (*p* < 0.01) and at the final follow-up (*p* < 0.01). The segmental angle was also restored 1 year postoperatively (*p* = 0.03). However, there was no significant difference in the segmental angle at the final follow-up (*p* = 0.10). As for the lumbar factors, there were no significant improvements in either SL (1 year, *p* = 0.08; final follow-up, *p* = 0.14) or LL (1 year, *p* = 0.55; final follow-up, *p* = 0.62). Additionally, there were no significant corrections in the sacropelvic profile with respect to pelvic incidence (1 year, *p* = 0.47; final follow-up, *p* = 0.48), pelvic tilt (1 year, *p* = 0.33; final follow-up, *p* = 0.45), sacral slope (1 year, *p* = 0.94; final follow-up, *p* = 0.79), and overall sagittal balance (1 year, *p* = 0.97; final follow-up, *p* = 0.80).

The clinical outcomes are summarized in Table 3. Regarding pain control, the VAS scores significantly decreased after 1 year (*p* < 0.01) and at the final follow-up (*p* < 0.01). Disability also improved during both evaluation periods (1 year, *p* = 0.01; final follow-up, *p* > 0.01). The rate of perioperative and early complications was 10.52% (*n* = 2) (incidental dura tear, 5.26% (*n* = 1) and wound dehiscence, 5.26% (*n* = 1)). All complications were treated conservatively. Related hematoma, infection, and reoperation were not identified. However, radiologic implant complications were identified in eight patients (42.10%), including subsidence in four patients (21.05%), additional ASD in three patients (15.79%), retropulsion in three patients (15.79%), cage breakage in one patient (5.26%), and pseudoarthrosis in one patient (5.26%). Three cases of combined retropulsion and subsidence, one case of additional ASD and subsidence, and one case of cage pseudoarthrosis and retropulsion were also reported.

## 4. Discussion

In this study, the clinicoradiologic outcomes of stand-alone posterior expandable cages for ASD were investigated. The results indicate that the stand-alone posterior expandable cage technique yielded favorable outcomes with respect to pain management, disability, and local restoration of segmental lordosis, without early complications and revision. However, there were no apparent long-term benefits for local restoration and sagittal balance. Furthermore, implant problems were highly frequent, leading to radiologic restoration failure.

ASD with LSCS is a common complication of spinal fusion surgery, occurring in 5.2–18.2% of patients [1,2]. The main purpose of indirect and direct interbody fusion is the stabilization and immobilization of each segment. However, this fixation consequently puts pressure on the upper and lower parts. These movement limitations accelerate the degeneration of the disc, ligamentum flavum, and posterior facet. In general, ASD treatment requires decompression of the narrowed spinal canal, restoration of sagittal balance, and stabilization of the interbody space. The previous operation site is a major factor to be considered in reoperation for ASD as injury to the previous operation site can result in dural tear and nerve injury [10]. Previous posterior screws and rods have to be removed, and patient management needs to be changed, including the ASD level.

There are several treatment approaches for ASD with LSCS. Extensive correction of sagittal balance and direct decompression are considered standard treatments for ASD because they can address three important conditions, namely, stenosis, kyphosis, and instability [11]. However, they are associated with prolonged operation time and risk of dura tear and infection [12]. Minimally invasive indirect interbody fusion including lateral interbody fusion and anterior fusion showed favorable outcomes in limited indications [13]. Further, there is minimal risk of vascular injury and retrograde ejaculation. However, indirect fusion cannot directly increase the area of the neural canal. Microscopic and endoscopic techniques for decompression only showed favorable short-term outcomes [14,15]. However, simple enlargement of the spine cannot restore sagittal balance; moreover, removal of the posterior laminofacet complex can accelerate the recurrence of stenosis [16]. Another minimally invasive option is an interbody spacer, but decompression of the spinal canal is limited with this procedure, and it is associated with a risk of disc space preservation [17].

**Figure 4:**
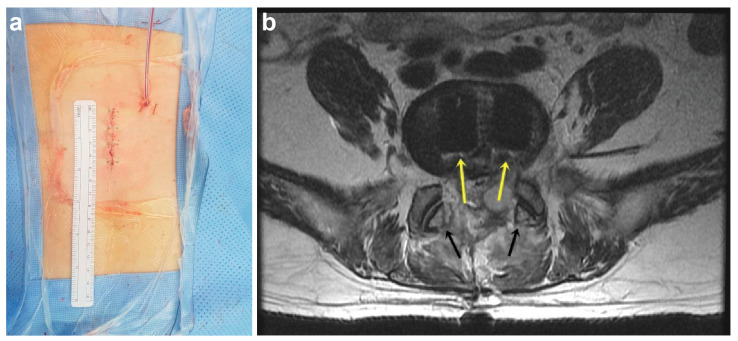
Postoperative outcome of the stand-alone cage technique. Incision size is <5 cm (**a**), with facet preservation (black arrows). Bilateral insertion (yellow arrows) (**b**).

Posterior expandable stand-alone cages are designed to restore 9° at each level with facet preservation (Figure 4a) while being minimally invasive (only a <5 cm incision is required, Figure 4b). This technique can also avoid injury to the previous operation site even with screw replacement and malpositioning. These advantages make it ideal for postoperative ASD. In contrast, recurrent disc herniation and calcified discs require wide dissection. ASD also requires interbody fusion, which can also be an indication for stand-alone cages. It was also found that augmentation due to osteoporotic compression fracture can achieve interbody fusion and decompression without the risk of screw placement. Previous studies have shown low rates of recurrent disc herniation [18] and spondylolisthesis [19].

Clinically, the present interbody technique achieved favorable pain and disability control without major complications. The rate of perioperative complications and reoperation was only 10.26% and 0%, lower than those reported in other revision procedures [20,21]. Radiologically, good local disc and foraminal height restoration were also achieved in both the short and long terms. However, the correction of sagittal balance was not achieved in either the short or long term. Moreover, there is a potential risk of incomplete decompression when partial facetectomy is performed. In addition, the high rates of subsidence, pseudoarthrosis, and implant breakage need to be addressed. The high subsidence rate may be due to the severe preoperative radiologic degeneration of the patients and may be associated with lower rates of LL restoration [22]. This could be solved with osteoporosis medication and improvement in the fusion material. In view of these results, this technique is beneficial for high-risk or elderly patients, whereas it has no apparent advantages in patients with severe kyphosis in sagittal balance requiring multilevel operations.

Implant, patient, and technical factors have to be balanced for better outcomes. First, the strength of the implant has to be improved. Without posterolateral fixation, the materials for interbody fusion need to be stronger. Aside from titanium, polyetheretherketone [23] and hydroxyapatite have also been used to develop cages [24]. Customized designs and individual clinical factors also need to be considered. Three-dimensional printed cages have been reported to show favorable outcomes [25]. Regarding patient factors, osteoporosis and vitamin D deficiency can cause insufficient fusion [26]. Thus, vitamin D supplementation, as well as teriparatide [27] and denosumab [28], should be considered as they can improve the fusion rate. Regarding technical factors, careful preparation and microscopic and endoscopic assistance, to avoid endplate destruction, are suggested. Proper allogenic and autologous grafts [29] also increase the fusion rate and preservation of restored sagittal balance. In the present study, a brace was applied to the patients for three months based on the features of ASD, but the duration could be reduced based on radiologic outcomes.

This study has limitations inherent to its retrospective, single-center design. The sample size was small, and comparisons with a control group and the application of widely used surgical techniques were lacking. A large-scale double-blind randomized control trial with a multicenter prospective design may better establish the reliability of this technique. In addition, this study used the cages for various types of ASD with LSCS, and more specific evaluations according to the type of ASD are needed. Despite these limitations, this study remains valuable because it provides evidence as to the clinicoradiologic benefits of the stand-alone posterior expandable cage technique as an initial procedure for postoperative ASD with LSCS.

## 5. Conclusions

A stand-alone expandable cage technique should only be considered for older adults and patients with previous extensive fusion. The technique is an effective modality for pain and disability control in ASD with LSCS; however, it does not correct the sagittal balance and spinopelvic parameters, and implant failure cannot be avoided. Moreover, the applicability of this technique for other pathological conditions remains questionable, and the strength of the cage material and subsidence require further improvement.

## Figures and Tables

**Table 1:** Patient characteristics.

Characteristics	Value
Female sex, *n* (%)	12 (63.16)
Age (years), mean ± SD	67.13 ± 9.31
Symptoms, *n* (%)	
Sensory	4 (21.05)
Sensorimotor	11 (57.89)
Bladder and bowel	4 (21.05)
ASA-PS grade, *n* (%)	
I	3 (15.79)
II	15 (78.95)
III	1 (5.26)
Initial operation, *n* (%)	
PLF	4 (21.05)
PLIF	9 (47.37)
Stand-alone cages	5 (26.32)
PVP	1 (5.26)
Involved levels, *n* (%)	
1	13 (68.42)
2	5 (26.31)
3	1 (5.26)
Duration of ASD (years), mean ± SD	8.26 ± 4.04
Follow-up duration (years), mean ± SD	7.61 ± 2.03
Inclusion of sacrum level, *n* (%)	1 (5.26)
Pfirrmann disc degeneration grade at the ASD level, *n* (%)	
4	6 (31.58)
5	13 (68.42)

Abbreviations: ASD, adjacent segment degeneration; SD, standard deviation; PLF, posterolateral fusion; PLIF, posterior lumbar interbody fusion; PVP, percutaneous vertebroplasty; ASA-PS, American Society of Anesthesiologists physical status.

**Table 2:** Radiologic outcomes of stand-alone posterior expandable cages for adjacent segment degeneration with lumbar spinal canal stenosis.

Variables		Preoperative	After 1-Year Follow-Up	*p*-Value	Final Follow-Up	*p*-Value
Local factors	Anterior disc height at the ASD level (mm), mean	7.38	14.51	<0.01 †*	13.36	<0.01 †*
	Middle disc height at the ASD level (mm), mean	6.01	11.93	<0.01 †*	11.22	<0.01 †*
	Posterior disc height at the ASD level (mm), mean	5.01	9.45	<0.01 †*	8.87	<0.01 †*
	Foraminal height at the ASD level (mm), mean	14.22	18.57	<0.01 †*	17.99	0.01 †*
	Segmental angle at the ASD level (°), mean	2.40	7.86	0.03 †*	6.53	0.10 †
Lumbar factors	Short lumbar lordosis (°), mean	11.64	18.36	0.08 †	17.34	0.14 †
	Whole lumbar lordosis (°), mean	27.12	30.36	0.55 †	29.80	0.62 †
Sacropelvic profiles	Pelvic incidence (°), mean	47.40	49.78	0.47 †	49.75	0.48 †
	Pelvic tilt (°), mean	23.23	26.38	0.33 †	25.71	0.45 ‡
	Sacral slope (°), mean	24.13	23.97	0.94 †	23.56	0.79 †
	C7 plumb line (cm), mean	4.94	4.00	0.97 †	4.21	0.80 †

† Univariate t-test, ‡ Mann–Whitney test. Segment degeneration: † unpaired t-test, ‡ Mann–Whitney test, * *p* < 0.05. Abbreviation: ASD, adjacent segment degeneration.

**Table 3:** Clinical outcomes of stand-alone posterior expandable cages for adjacent segment degeneration with lumbar spinal canal stenosis.

Variables	Preoperative	1-Year Follow-Up	*p*-Value	Final Follow-Up	*p*-Value
Pain (VAS, mean)	6.78	2.26	<0.01 †*	2.12	<0.01 †*
Disability (ODI, mean)	28.89	11.96	<0.01 †*	11.26	<0.01 †*
Bone fusion grade	Grade 1, 2 (achieved fusion, *n* (%))	16 (84.21)			
	Grade 3 (non-union, *n* (%))	2 (10.52)			
	Grade 4 (pseudoarthrosis, *n* (%))	1 (5.26)			
Early complication rate	Total	2 (10.52)			
	Incidental dura tear	1 (5.26)			
	Wound dehiscence	1 (5.26)			
Implant problems	Total	8 (42.10)			
	Retropulsion (*n*), (%)	3 (15.79)			
	Subsidence (*n*), (%)	4 (21.05)			
	Pseudoarthrosis (n), (%)	1 (5.26)			
	Cage breakage (*n*), (%)	1 (5.26)			
	Adjacent segment degeneration (*n*), (%)	3 (15.79)			

† Unpaired *t*-test, * *p* < 0.05. Abbreviations: VAS, visual analogue scale; ODI, Oswestry Disability Index.

## Data Availability

Data available in a publicly accessible repository.
